# DISABILITY IN PRISON ACTIVITIES OF DAILY LIVING AND DEPRESSION IN OLDER PRISONERS: A PROSPECTIVE STUDY

**DOI:** 10.1093/geroni/igz038.2618

**Published:** 2019-11-08

**Authors:** Lisa C Barry, Dorothy Wakefield, David C Steffens, Yeates Conwell

**Affiliations:** 1 University of Connecticut School of Medicine, Farmington, Connecticut, United States; 2 UCONN Center on Aging, Farmington, Connecticut, United States; 3 University of Rochester School of Medicine, Rochester, New York, United States

## Abstract

The U.S. prison population is aging; more persons are being incarcerated in the second half of life and are aging “in place.” In the first prospective study to evaluate older prisoners’ mental health (Aging INSIDE), we determined if disability in activities of daily living specific to prison, prison activities of daily living (PADLs), predicts depression in this vulnerable population. To date, 134 older prisoners (age ≥50) sentenced at 9 Connecticut correctional facilities completed in-person interviews (baseline and one-year follow-up). A score of ≥10 on the 9-item Physician Health Questionnaire (PHQ-9) indicated depression. Participants were considered to have PADL disability if they reported any of the following as “very difficult” or “cannot do”: climbing on/off the top bunk (34%), cleaning their cell (5%), hearing orders (6%), walking while wearing handcuffs (33%) or shackles (34%), standing in line for medications (4%), and walking to chow (5%). Participants were mean age 57.0±6.6 years (range 50-79 years), racially diverse (43% White, 38% Black, 19% Hispanic/Other), 69 (50%) had PADL disability, and 35 (25%) were depressed at follow-up. Using logistic regression and controlling for gender, number of chronic conditions, lifetime suicide attempt, and baseline depression, baseline PADL disability was associated with depression one year later (OR = 3.41; 95%CI = 1.16, 9.97). As depression is a strong risk factor for subsequent suicide, and given the high rate of suicide among older prisoners in the U.S., these preliminary results indicate that PADL assessment may offer a simple means of identifying older prisoners at risk of depression.

